# The diagnostic and immunomodulatory role of IL-37 in pediatric sepsis

**DOI:** 10.3389/fimmu.2026.1765956

**Published:** 2026-02-12

**Authors:** Jin Ma, Yanling Zhang, Changrui Sun, Hao Yang, Zhixin Song, Feiqi Huang, Chunxiang Wu

**Affiliations:** 1 Department of Laboratory Medicine and Sichuan Provincial Key Laboratory for Human Disease Gene Study, Sichuan Provincial People’s Hospital, University of Electronic Science and Technology of China, Chengdu, China; 2 Department of Gastrointestinal Surgery, Sichuan Provincial People’s Hospital, University of Electronic Science and Technology of China, Chengdu, China; 3 Department of Medicine, Sichuan Provincial People’s Hospital, University of Electronic Science and Technology of China, Chengdu, China; 4 Department of Clinical Laboratory, Children’s Hospital of Chongqing Medical University, Chongqing, China; 5 Department of Pediatrics, Sichuan Provincial People’s Hospital, University of Electronic Science and Technology of China, Chengdu, China

**Keywords:** diagnostic biomarker, immunotherapy, interleukin-37, macrophage cells, pediatric sepsis

## Abstract

**Objective:**

Sepsis arises from a dysregulated host inflammatory response to infection. The levels and pathogenic role of interleukin-37 (IL-37) in pediatric sepsis remain to be fully elucidated.

**Methods:**

Serum IL-37 concentrations were measured in two independent cohorts of pediatric patients with sepsis from Chongqing (discovery cohort, n=40) and Sichuan (validation cohort, n=105). The immunomodulatory effects of IL-37 were systematically investigated through: 1) a murine sepsis model (cecal ligation and puncture), and 2) ex vivo experiments using peripheral blood mononuclear cells (PBMCs) from patients under standardized culture conditions.

**Results:**

Admission serum IL-37 levels were significantly higher in pediatric patients with sepsis compared to non-septic patients and healthy controls. To assess its diagnostic potential, ROC curve analyses were performed, yielding areas under the curve (AUC) of 0.76 [P<0.0001; 95% confidence interval (95% CI), 0.66 - 0.85] and 0.77 [P<0.0001; 95% CI, 0.71 - 0.84] from the two medical centers. In septic mice, therapeutic administration of recombinant IL-37 significantly attenuated systemic inflammation (reduced IL-6, CXCL-1, CCL-2; increased IL-10), decreased the proportion of M1 macrophages without altering total macrophage counts in peritoneal lavage fluids (PLF), and improved survival rates. *In vitro*, IL-37 neutralization with a specific antibody in septic PBMCs increased the percentages of CD4+ T cells and NKT cells.

**Conclusion:**

This study identifies elevated IL-37 as a potential diagnostic biomarker for pediatric sepsis and demonstrates its role in modulating hyperinflammation and immune cell differentiation. IL-37 represents a promising therapeutic target for pediatric sepsis.

## 1 Introduction

Sepsis is a life-threatening organ dysfunction resulting from a disordered host response to infection, as defined in adults by the Third International Consensus Definitions for Sepsis and Septic Shock (Sepsis-3) (1). Recently, the 2024 International Consensus on Sepsis and Septic Shock in Children updated pediatric-specific definitions, drawing upon the 2016 adult criteria (2). The global burden of sepsis remains substantial, with an estimated 48.9 million cases worldwide and over 11.0 million sepsis-related deaths annually (3). Approximately 3.0 million cases occur in neonates and 1.2 million in children, with mortality rates for pediatric sepsis ranging from 4% to 50% (4–7). Consequently, the WHO resolution on sepsis emphasizes the need for enhanced diagnosis, prevention, and treatment strategies (8, 9).

Poor outcomes in sepsis management are often attributed not to resource limitations but to delays in the early recognition and treatment of the condition (10). Diagnosing sepsis and assessing its severity is complicated by the highly variable and non-specific nature of its signs and symptoms (11). Although biomarkers such as C-reactive protein (CRP) and procalcitonin (PCT) have been utilized in clinical practice for many years and are included in various global clinical prediction tools, their non-specificity limits their utility (12). A critical aspect of successful sepsis treatment is the prompt removal of the source of infection (13). In pediatric clinical practice, the challenge lies in diagnosing sepsis as early as possible to maximize survival rates among affected children. This necessitates the exploration of new diagnostic targets and a comprehensive understanding of the complex immunopathological mechanisms regulating the host’s inflammatory response during sepsis. Cytokines and chemokines play pivotal roles in mediating both inflammatory and anti-inflammatory responses in sepsis (14), offering new possibilities for immune-based diagnostic and therapeutic interventions. Gaining insights into the complex interactions among these immune mediators within the sepsis framework may pave the way for novel strategies for diagnosing and treating pediatric patients.

Interleukin-37 (IL-37), classified under the IL-1 cytokine family and previously referred to as IL-1F7 (15, 16), has been recognized as a potent immunomodulator. The gene responsible for IL-37 is located on chromosome 2q12-13 (17), and five splice variants (IL-1F7a-e) have been identified (18), with IL-1F7b being the most abundant. IL-37a has demonstrated greater efficacy than IL-37b in protecting against Toll-like receptor-induced cytokine hypersecretion and lethal endotoxic shock (19). This interleukin significantly influences both innate and acquired immunity and exhibits anti-inflammatory properties in conditions such as inflammatory bowel disease and allergic contact dermatitis (20, 21). Its role in bone metabolism also indicates potential therapeutic avenues for inflammatory bone disorders (22). However, investigations regarding IL-37 in sepsis patients remain scarce. Wang et al. documented increased IL-37 levels in adults suffering from sepsis (23). Furthermore, our earlier research suggested that IL-37 could serve as a predictor of mortality risk in adult sepsis patients (16). Nonetheless, the role of IL-37 in pediatric sepsis—a population with distinct immunological and developmental characteristics—has yet to be established. In this study, we conducted a translational investigation combining clinical cohorts, animal models, and *in vitro* assays. Our research aims to evaluate the role of IL-37 in pediatric sepsis by achieving three objectives (1): validating IL-37 as an early diagnostic biomarker across two independent cohorts; (2) elucidating its immunomodulatory effects *in vivo*, specifically regarding inflammation regulation and its impact on macrophages; and (3) illustrating its effects on T cell subsets *in vitro*, which were observed through the administration of peripheral blood mononuclear cells (PBMC).

## 2 Materials and methods

### 2.1 Pediatric patients and controls

We conducted a prospective cohort study across two centers involving pediatric patients diagnosed with sepsis. Subjects were recruited from the Children’s Hospital of Chongqing Medical University (Chongqing, China) and the Sichuan Provincial People’s Hospital, University of Electronic Science and Technology of China (Sichuan, China).

Inclusion Criteria: 1) Pediatric patients diagnosed with sepsis, based on the International Consensus Criteria for Pediatric Sepsis and Septic Shock. Specifically, the Phoenix Sepsis Criteria require the presence of a suspected infection and Phoenix Sepsis Score≥2. The Phoenix Sepsis Score assesses damage to the respiratory, cardiovascular, coagulation, and/or neurological systems. Septic shock is defined as sepsis accompanied by at least one cardiovascular point on the Phoenix Sepsis Score (2). The PSS is a clinical tool designed to aid in the early identification of sepsis in patients. It was originally validated in a large cohort study, demonstrating its effectiveness in predicting sepsis-related outcomes. 2) The study participants consisted of children aged over 1 month and under 18 years.

Exclusion Criteria: 1) Patients with missing or incomplete key clinical data. 2) Patients with pediatric stays shorter than 24 hours. 3) Parameters for exclusion included HIV infection, malignant tumors, and the use of immunosuppressive therapies within 2 weeks prior to participation, as well as refusal of informed consent by family members. Concurrently, 32 age- and sex-matched non-septic patients with infections were recruited as pediatric non-septic patients from the medical examination center of the Children’s Hospital of Chongqing Medical University. Additionally, 32 age- and sex-matched healthy children were enrolled from the same center as healthy controls; all healthy donors were free of medical issues. To further validate our findings, an independent cohort of 105 pediatric patients with sepsis, 40 non-septic pediatric patients with infections, and 48 healthy controls were recruited from Sichuan Provincial People’s Hospital, University of Electronic Science and Technology of China, following the same inclusion and exclusion criteria as in the discovery cohort. Detailed diagnoses of non-septic patients with infections from both centers are listed in **Table 1** and **Table 2**.

**Table 1:** Characteristics of pediatric patients with sepsis and control individuals in the discovery (Chongqing) cohort.

Characteristics	Healthy controls (n=32)	Non-sepsis patients(n=32)	Pediatric patients with sepsis(n=40)
Sex (Male/ Female)	22/10	25/7	34/6
Age(mouths)	11.5(8-35.25)	9.5(6-48)	7(2-12)
WBC (×10^9^)	6.82 (5.94-9.00)	9.18(7.73-13.90)	11.09(7.52-15.01)
CRP, mg/L	1.47(0.52-3.51)	31.81(13.35-67.48)	43.5(9.00-66.75)
IL-6, pg/ml	9.26(7.31-34.75)	38.22(8.4-47.67)	43.35(36.42-50.55) ^*^
PCT, ng/ml	0.27(0.11-0.91)	0.19(0.10-0.65)	5.30(0.35-22.61) ^****^
Infection site, number of patients(case)			
Respiratory	NA	17	19
Urinary	NA	4	4
Abdominal	NA	3	1
Other	NA	8	16
Isolates, number of patients (case)			
Gram positive	NA	1	7
Gram negative	NA	1	10
Other microbial infections	NA	NA	9 (Fungus:7 Virus:2)

Data are expressed as median (interquartile range), WBC, white blood cells; CRP, C-reaction protein; PCT, Procalcitonin; NA, not applicable. * P<0.5, ****P<0.0001 Compared with pediatric non-sepsis patients.

**Table 2:** Characteristics of pediatric patients with sepsis and control individuals in the validation (Sichuan) cohort.

Characteristics	Healthy controls (n=48)	Non-sepsis patients(n=40)	Pediatric patients with sepsis(n=105)
Sex (Male/ Female)	30/18	19/21	57/48
Age(mouths)	36(16-81)	48(30.5-72)	36(26-60)
WBC (×10^9^)	6.44(5.49-7.54)	10.85(6.79-17.76)	16.24(8.63-22.27) ^*^
CRP, mg/L	5.7(2.38-24.87)	55.62(23.57-81.48)	76.23(36.43-112.00)^*^
IL-6, pg/ml	8.50(6.98-69.49)	39.64(21.15-65.79)	77.74(44.55-217.63)^****^
PCT, ng/ml	0.055(0.05-0.23)	0.38(0.21-0.81)	1.24(0.47-3.58) ^****^
Infection site, number of patients(case)			
Respiratory	NA	29	73
Urinary	NA	2	4
Abdominal	NA	4	7
Other	NA	5	21
Isolates, number of patients (case)			
Gram positive	NA	1	17
Gram negative	NA	NA	7
Other microbial infections	NA	Virus:1	5(Mycoplasma: 2 Virus:3)

Data are expressed as median (interquartile range), WBC, white blood cells; CRP, C-reaction protein; PCT, Procalcitonin; NA, not applicable. *P<0.05 ****P<0.0001 Compared with pediatric non-sepsis patients.

Venous blood samples were collected from all subjects on the enrollment day, with serum separated via centrifugation at 3500 RPM for 10 minutes. The samples were subsequently frozen at -80°C until testing for interleukin-37 (IL-37) and interleukin-6 (IL-6). Additionally, a 5 ml sample of peripheral blood was subjected to Ficoll density gradient centrifugation to extract peripheral blood mononuclear cells (PBMCs) for *in vitro* cell culture experiments. Data pertaining to the enrolled patients along with fundamental clinical information were gathered. All laboratory assessments, including enzyme-linked immunosorbent assay (ELISA) and flow cytometry, were conducted by technicians unaware of the clinical categorization.

### 2.2 Cytokines and chemokines

The concentrations of IL-37 (Catalog No: DY1975-05, Lot No: P325850, R&D Systems, USA) and IL-6 (Catalog No: DY206-05, Lot No: P326914, R&D Systems, USA) in pediatric sepsis patients were measured using ELISA.

### 2.3 Animal models and treatment

C57BL/6 mice, aged 6 to 8 weeks and weighing approximately 21 g ± 0.5 g, were acquired and bred in specific pathogen-free conditions at the Animal Center of Sichuan Provincial People’s Hospital, University of Electronic Science and Technology of China.

#### 2.3.1 Mouse model of cecal ligation and puncture

To induce polymicrobial sepsis, C57BL/6 mice were housed in a specific pathogen-free environment for one week prior to undergoing cecal ligation and puncture (CLP), as previously described in the literature (24–26). Mice were anesthetized intraperitoneally with a mixture of xylazine (4.5 mg/kg) and ketamine (90 mg/kg) for analgesia. A midline incision was performed in the anterior abdomen to access the cecum, which was ligated at its external third and subsequently punctured with a 22-gauge needle, while a 21-gauge needle was used to puncture the external two-thirds (severe CLP, with mortality rates ranging from 70% to 100%). After the procedure, the cecum was carefully returned to the peritoneal cavity, and the incisions were sutured. Mice that underwent sham operations served as the surgical control group, following identical procedural steps but without cecum ligation or puncture (24, 27). At the experiment’s endpoint, animals were euthanized via cervical dislocation.

#### 2.3.2 *In vivo* administration of recombinant human IL-37

Recombinant human IL-37 protein (Lot: MLB0319061, R&D Systems, USA) was administered intraperitoneally at a dose of 2 µg per injection (28, 29) immediately following the CLP procedure. Phosphate-buffered saline (PBS) was used as the control vehicle. In the CLP sepsis model, which consistently yields mortality rates between 70% and 100%, the administration of rhIL-37 (2 µg per injection) at the time of CLP was found to significantly affect mouse survival.

#### 2.3.3 Peritoneal lavage fluid cytokine and chemokine analysis and cytology assessment

To analyze chemokines and cytokines, PLF was obtained using 5 ml of PBS, followed by centrifugation, to isolate the acellular supernatant, which was preserved at -80°C. The levels of IL-6, chemokine (C-X-C motif) ligand 1 (CXCL1), CCL2 (chemokine CC receptor ligand 2), and interleukin-10 (IL-10) in both PLF and serum from CLP mice were assessed using ELISA (IL-6 Cat: 431304, Lot: B358226; CXCL1 Cat: 447504, Lot: B376252; CCL2 Cat: 432704, Lot: B367248; IL-10 Cat: 31414, Lot: B366045, BioLegend, San Diego, CA), adhering to the manufacturer’s guidelines.

The supernatant obtained was utilized for cytokine analysis, while the cell pellet was re-suspended in 3 ml of PBS for flow cytometry analysis and cytological evaluation. Glass slides for cell spinning were prepared using a film spinner and subsequently stained with Wright-Giemsa stain for observation.

#### 2.3.4 Flow cytometry for PLF macrophage detection

Cells from the PLF were identified using specific monoclonal antibodies against CD45-APC-R700 (BD Pharmingen™, clone 30-F11), CD11b-FITC, lymphocyte antigen 6 complex, locus G (Lg6G-BV421, BD Pharmingen™, clone RB6-8C5), F4/80-PE (BD Pharmingen™, clone T45-2342), CD86-PE-CY7 (BD Pharmingen™, clone PO3), and CD206-APC (BD Pharmingen™, clone Y17-505), as previously described [24]. The detailed procedure is as follows: 100 µl of red blood cell lysate was added for 5 minutes, followed by 200 µl of PBS. The solution was then centrifuged at 850 x g for 5 minutes at 4°C. Following this, the supernatant was removed, and the cells were recovered. Lastly, 100 µl of PBS was added to re-suspend the cells. After this step, 2 µl of antibodies including mouse CD45-APC-R700, CD11b-FITC, F4/80-PE, CD86-PE, and CD206-APC were introduced, and the samples were incubated for 30 minutes after blocking non-specific binding sites. The flow cytometry samples prepared were subsequently analyzed through the BD-FACS Diva System, with data being processed via FlowJo Version 10.0.

### 2.4 *In vitro* culture of peripheral blood mononuclear cells

PBMCs were isolated from pediatric sepsis patients. The recombinant human IL-37 protein (1 µg, 10 ng; Lot: MLB0319061, R&D Systems, USA) and anti-IL-37 (1 µg, 10 ng; Lot: UAE0322071, R&D Systems, USA) were added to the PBMC cultures, which were maintained in Gibco 1640 medium supplemented with 10% fetal bovine serum at 37°C in a 5% CO_2_ atmosphere, with a cell density of 1×10^6/mL. Phosphate-buffered saline (PBS) served as the control. Following a 24-hour incubation, supernatants were collected for cytometric bead array analysis, and the cells were harvested for flow cytometry analysis. The cell suspension was labeled with the following antibodies: CD19-FITC (BECKMAN COULTER, clone J3-119, USA), CD3-PE (BECKMAN COULTER, clone UCHT1, USA), CD16-ECD (BECKMAN COULTER, clone 3G8, USA), CD56-ECD (BECKMAN COULTER, clone N901, USA), CD4-PerCP5.5 (BECKMAN COULTER, clone 13B8.2, USA), CD45-CY7 (BECKMAN COULTER, clone J33, USA), and CD8-APC (BECKMAN COULTER, clone B9.11, USA). Cellular immune function was assessed via flow cytometry using the Beckman Coulter Navios system, with data processed through Kaluza System software.

### 2.5 Statistical analysis

For statistical analysis, human data were represented as scatter dot plots illustrating medians and interquartile ranges, while mouse data were depicted in violin plots and scatter dot plots showing medians along with interquartile ranges. The Mann–Whitney U test was utilized for group comparisons. Survival analysis was conducted using the Kaplan–Meier method, with differences between groups assessed using the log-rank test. All statistical analyses were performed using GraphPad Prism version 9.3.0 (GraphPad Software, San Diego, CA). P-values less than 0.05 were deemed statistically significant.

## 3 Results

### 3.1 Elevated IL-37 levels in pediatric patients with sepsis

**Figure 1:**
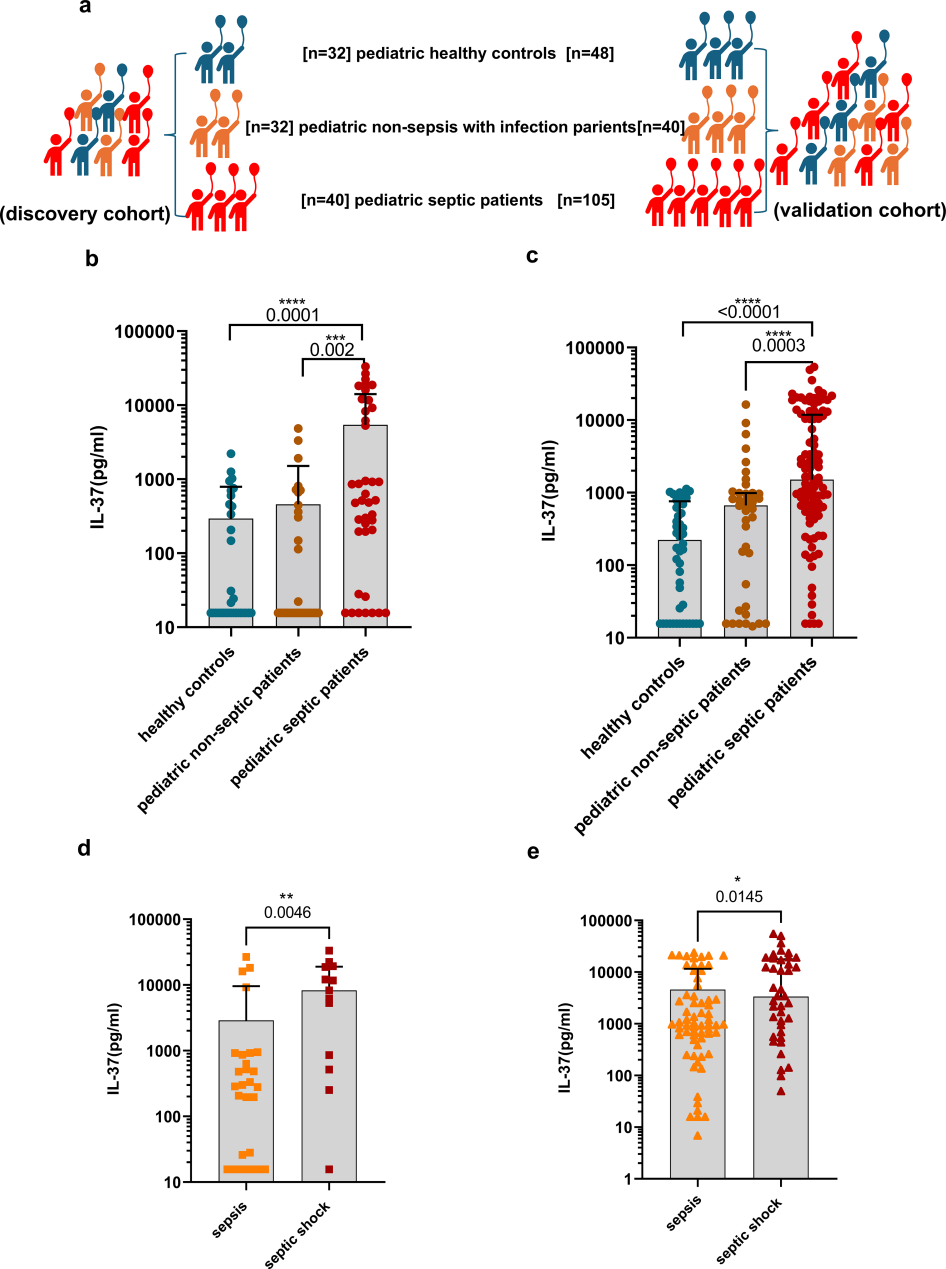
Serum IL-37 levels on the day of admission were elevated in pediatric patients with sepsis. Serum IL-37 concentrations were quantified using ELISA in peripheral blood samples. **(a)** Cohorts Design: The study included a discovery cohort-Chongqing comprising healthy controls (n = 32), pediatric non-septic infection controls (n = 32), and pediatric sepsis patients (n = 40), as well as a validation cohort consisting of healthy controls (n = 48), pediatric non-septic infection controls (n = 40), and pediatric sepsis patients (n = 105). **(b)** In the discovery cohort (Chongqing), serum IL-37 concentrations were significantly higher in pediatric patients with sepsis. **(c)** In the validation cohort (Sichuan), serum IL-37 concentrations were also elevated in pediatric patients with sepsis. **(d)** In the discovery cohort (Chongqing), serum IL-37 levels in children with septic shock were higher than those in children with sepsis without shock. **(e)** In the validation cohort (Sichuan), serum IL-37 levels in children with septic shock were higher than those in children with sepsis without shock. Statistical significance was observed with *P<0.05; **P<0.01; ***P<0.001; and ****P<0.0001.

**Figure 2:**
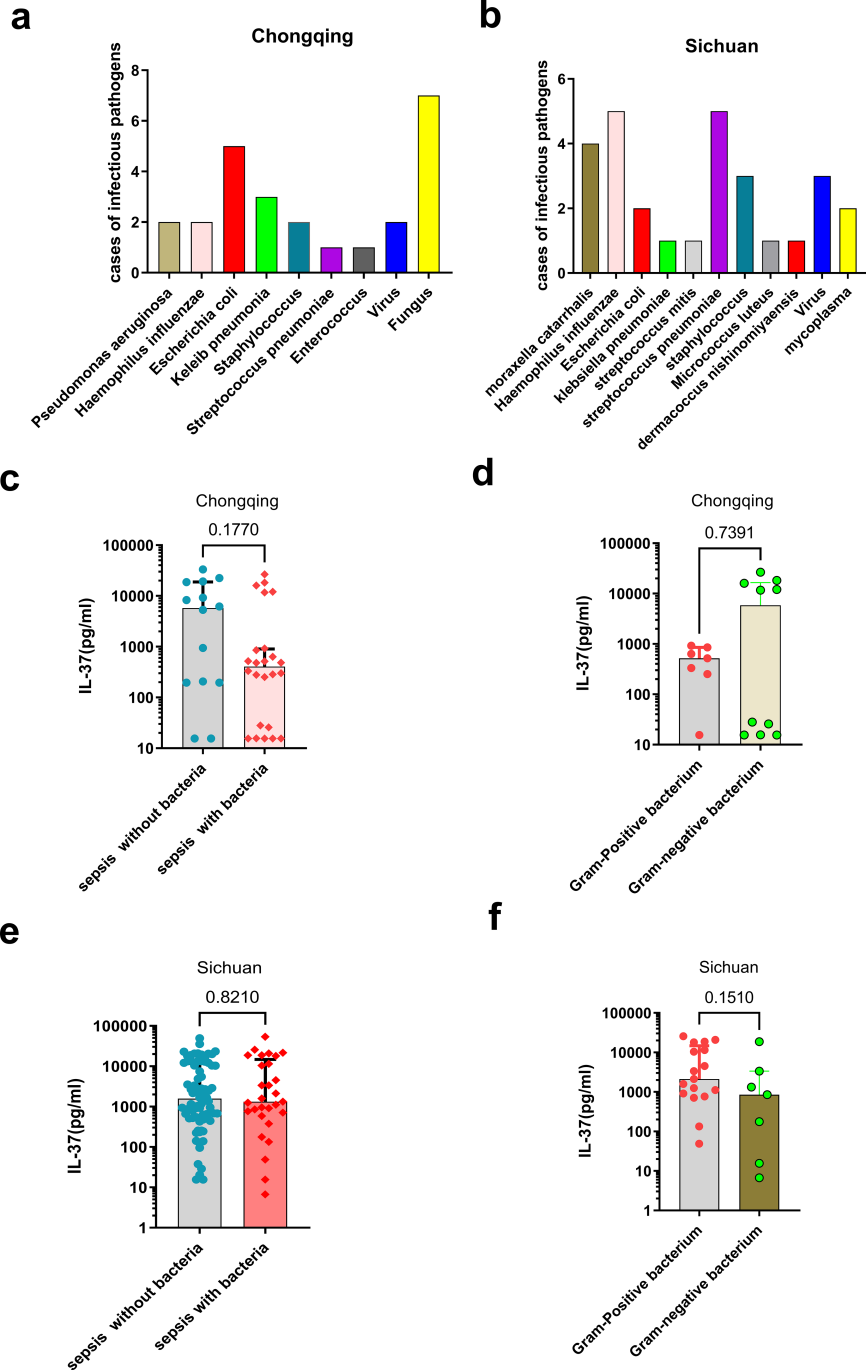
The level of IL-37 in pediatric patients with bacterial sepsis. The pathogenic bacteria infected in patients with sepsis, **(a)** in the discovery cohort (Chongqing), **(b)** in the validation cohort (Sichuan). Serum concentrations of IL-37 in patients with sepsis associated with bacterial infections were measured **(c)** in the discovery cohort (Chongqing) and **(e)** in the validation cohort (Sichuan). The serum IL-37 concentrations were evaluated in cases of sepsis caused by Gram-positive and Gram-negative bacteria **(d)** in the discovery cohort (Chongqing) and **(f)** in the validation cohort (Sichuan).

The research cohorts are illustrated in **Figure 1a**. The clinical features and demographic data of pediatric patients with sepsis, non-sepsis patients, and healthy controls are summarized in **Table 1** (discovery cohort from Chongqing) and **Table 2** (validation cohort from Sichuan). We assessed IL-37 concentrations in a cohort of pediatric sepsis patients from the Children’s Hospital of Chongqing Medical University. Serum IL-37 levels were significantly higher in the sepsis group compared to both pediatric non-sepsis patients and healthy controls (**Figure 1b**). To validate these findings, we measured IL-37 levels in an independent cohort of pediatric sepsis patients from Sichuan Provincial People’s Hospital, University of Electronic Science and Technology of China. Consistently, serum IL-37 concentrations were significantly elevated in 105 pediatric sepsis patients relative to 40 non-sepsis patients and 48 healthy controls (**Figure 1c**). Furthermore, in both the discovery cohort (**Figure 1d**) and validation cohort (**Figure 1e**), the serum IL-37 levels in children with septic shock were higher than those in children with sepsis without shock. The distribution of pathogenic bacteria identified in the pediatric sepsis patients is depicted in **Figure 2a** and **2b**. Notably, IL-37 levels did not differ significantly between patients with bacterial and non-bacterial causes of sepsis (**Figures 2c, e**). Similarly, no significant differences in IL-37 levels were observed between children with sepsis caused by Gram-positive bacteria and those caused by Gram-negative bacteria (**Figures 2d, f**).

### 3.2 Diagnostic potential of IL-37 in pediatric sepsis

**Figure 3:**
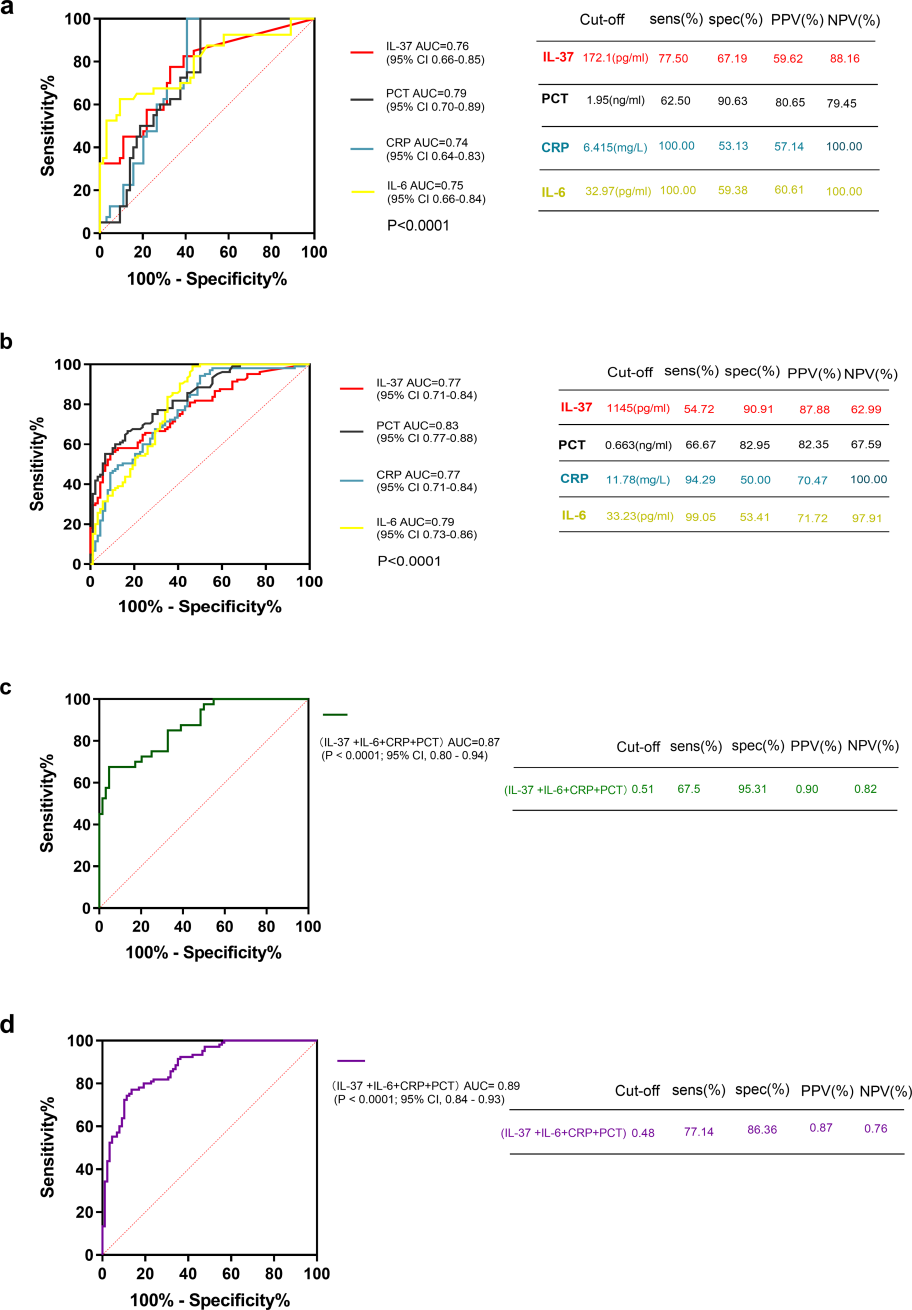
The ROC curves for IL-37 at admission for the diagnosis of pediatric patients with sepsis. **(a)** The ROC curve for IL-37, IL-6, CRP, and PCT at admission in the discovery cohort, which included 40 pediatric patients with sepsis from Chongqing. **(b)** The ROC curve for IL-37, IL-6, CRP, and PCT at admission in the validation cohort, which comprised 105 pediatric patients with sepsis from Sichuan. **(c)** The ROC curve for IL-37 combined with PCT, CRP, and IL-6 in the discovery cohort from Chongqing. **(d)** The ROC curve for IL-37 combined with PCT, CRP, and IL-6 in the validation cohort from Sichuan. AUC refers to the area under the ROC curve; the cut-off value indicates the optimal diagnostic cutoff points for pediatric patients with sepsis; Sens (%): sensitivity; Spec (%): specificity; PPV(%): positive predictive value; NPV(%): negative predictive value.

The diagnostic value of serum IL-37 in children with sepsis was evaluated through receiver operating characteristic (ROC) curve analysis. In the discovery cohort, the serum IL-37 concentration yielded an Area Under the Curve (AUC) of 0.76 (P < 0.0001; 95% confidence interval [95% CI], 0.66 - 0.85) for diagnosing sepsis in pediatric patients (**Figure 3a**). Using a serum IL-37 level of 172.1 ng/mL as the cutoff value, the sensitivity for diagnosing sepsis was 77.5%, while the specificity was 67.19%. This diagnostic performance was corroborated in the validation cohort, which demonstrated an AUC of 0.77 (P < 0.0001; 95% CI, 0.71 - 0.84) (**Figure 3b**). With a serum IL-37 level of 1145.0 ng/mL as the threshold value, the sensitivity for diagnosing sepsis in pediatric patients was 54.72%, and the specificity was 90.91%.

To further explore its diagnostic capacity in sepsis, IL-37 was combined with established biomarkers, including procalcitonin (PCT), C-reactive protein (CRP), and interleukin-6 (IL-6). In the discovery cohort, the combination of IL-37, PCT, CRP, and IL-6 significantly improved the AUC from 0.76 (for IL-37 alone) to 0.87 (P < 0.0001; 95% CI, 0.80 - 0.94) (**Figure 3c**). Analogously, in the validation cohort, the combined biomarker panel increased the AUC from 0.77 to 0.89 (P < 0.0001; 95% CI, 0.84 - 0.93) (**Figure 3d**).

### 3.3 IL-37 mitigates systemic inflammation and improves prognosis in mice with sepsis

**Figure 4:**
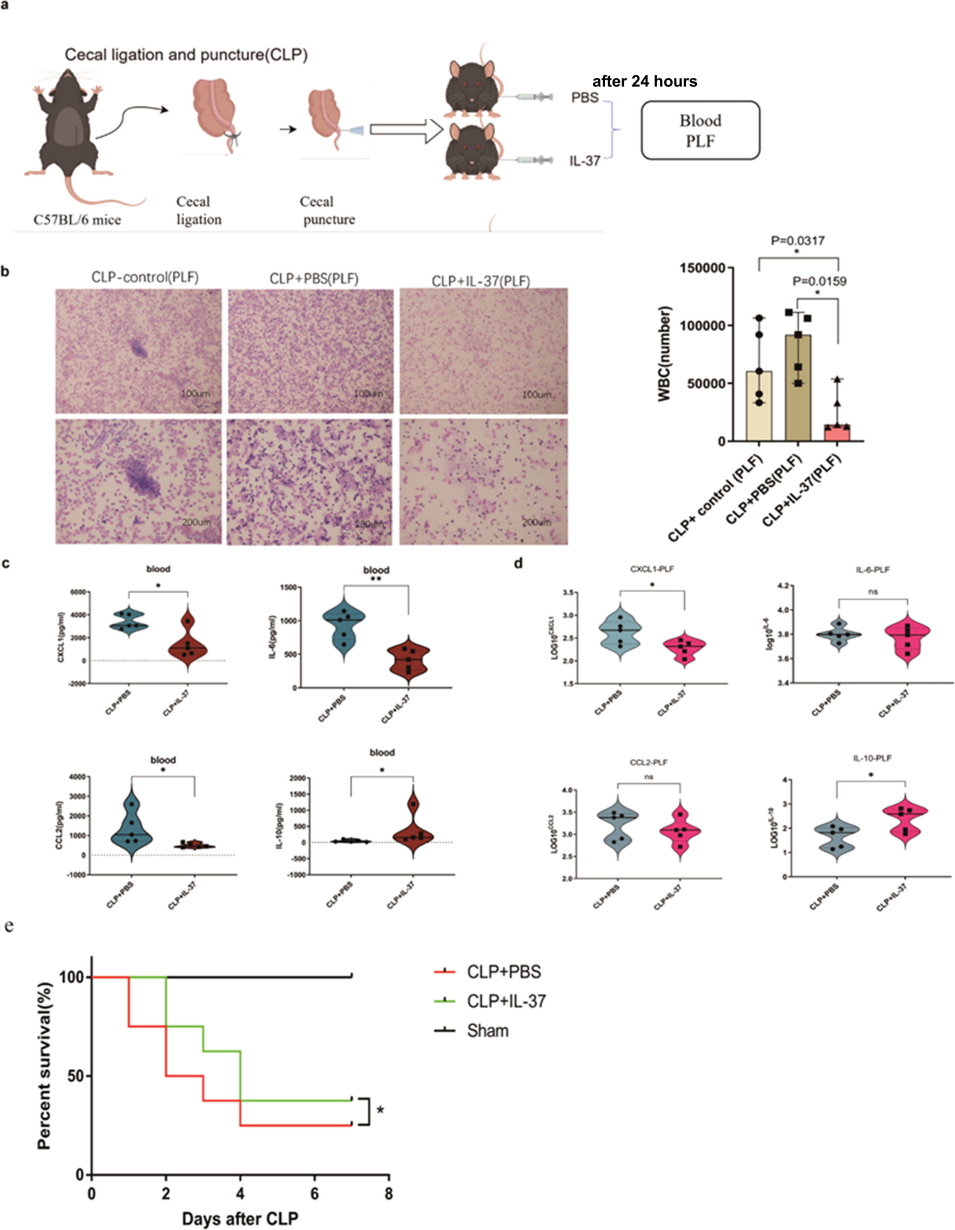
The involvement of IL-37 in regulating inflammation during sepsis *in vivo*. **(a)** The flow chart detailing the animal experiments was provided. **(b)** A significant decrease in white blood cell (WBC) count was observed in the peritoneal lavage fluid (PLF) 24 hours after the administration of IL-37, in comparison to the control group treated with PBS and the control following cecal ligation and puncture (CLP). The concentrations of cytokines and chemokines in **(c)** blood and **(d)** PLF from septic mice (n = 5 per group), treated with either IL-37 or PBS 24 hours post-CLP, were quantified using ELISA. **(e)** mice (n = 8 per group) were injected intraperitoneally with rhIL-37 (2 μg/injection) at CLP, and survival was monitored for 7 days. The results for CXCL-1, IL-6, CCL2, and IL-10 in PLF were presented in a logarithmic scale. Statistical significance was observed with *P<0.05; **P<0.01.

To further evaluate the therapeutic potential of IL-37, we developed a cecal ligation and puncture (CLP) sepsis model in mice and initiated an experiment involving the administration of IL-37 (**Figure 4a**). Mice treated with recombinant human IL-37 (2 µg) exhibited significantly lower serum concentrations of pro-inflammatory mediators, including IL-6, CXCL-1, and CCL-2, alongside elevated levels of the anti-inflammatory cytokine IL-10, compared to PBS-treated controls (**Figure 4c**). Furthermore, IL-37 treatment significantly enhanced the 7-day survival rates when compared to the PBS-treated group (Log-rank test, p < 0.05; **Figure 4e**).

### 3.4 IL-37 modulates the inflammatory response in PLF

Analysis of PLF indicated that IL-37 administration mitigated local inflammation. At 24 hours post-treatment, IL-37-treated sepsis mice demonstrated a reduction in total white blood cell (WBC) count and inflammatory cell infiltration compared to both PBS-treated and untreated CLP controls (**Figure 4b**). Additionally, the concentration of the chemokine CXCL-1 in PLF was decreased, while levels of the anti-inflammatory cytokine IL-10 were significantly elevated. No notable differences were observed in the concentrations of IL-6 and CCL-2 within the PLF (**Figure 4d**).

### 3.5 IL-37 regulates peritoneal macrophage polarization

**Figure 5:**
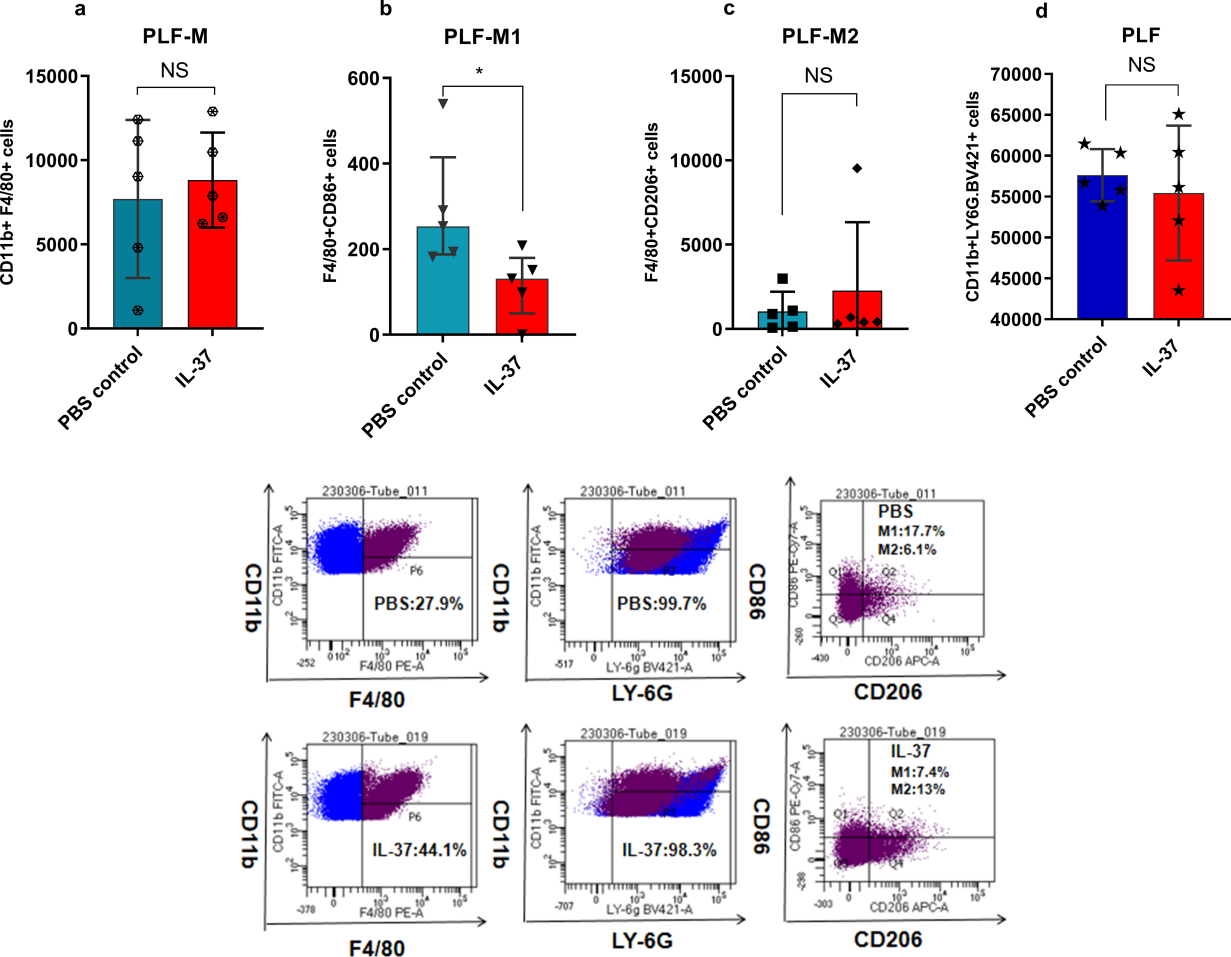
The effects on macrophages and neutrophils in the peritoneal lavage fluid (PLF) 24 hours post-treatment with IL-37 or PBS in CLP mice (n=5 per group). Peritoneal macrophages were identified by gating on CD11b+F4/80+ cells. M1 macrophages in the abdominal cavity were identified by gating on F4/80+CD86+, while M2 macrophages were identified by gating on F4/80+CD206+. Peritoneal neutrophils were identified by gating on CD11b+Ly6G+ cells. **(a)** Shows the total number of macrophages in the PLF of CLP mice treated with IL-37–24 hours post-CLP. **(b)** Displays the number of F4/80+CD86+(M1) macrophages in the PLF of CLP mice treated with IL-37–24 hours post-CLP. *P < 0.05 indicates a significant difference when compared to septic mice treated with PBS control (Mann-Whitney U test). **(c)** Illustrates the number of F4/80+CD206+(M2) macrophages in the PLF of CLP mice treated with IL-37–24 hours post-CLP. **(d)** Depicts the number of neutrophils in the PLF of CLP mice treated with IL-37–24 hours post-CLP.

Flow cytometric analysis of PLF from CLP mice indicated no significant difference in the proportions of total macrophages or neutrophils between the IL-37-treated and PBS-treated groups. However, detailed immunophenotyping revealed a specific alteration in macrophage subsets: while the percentage of M2 macrophages (defined as F4/80+ CD11b^+^ CD206^+^) remained unchanged, a significant reduction in the proportion of M1 macrophages (F4/80+ CD11b+ CD86+) was observed following IL-37 treatment (**Figure 5**).

### 3.6 Impact of IL-37 neutralization on immune cell distribution in pediatric sepsis *in vitro*

**Figure 6:**
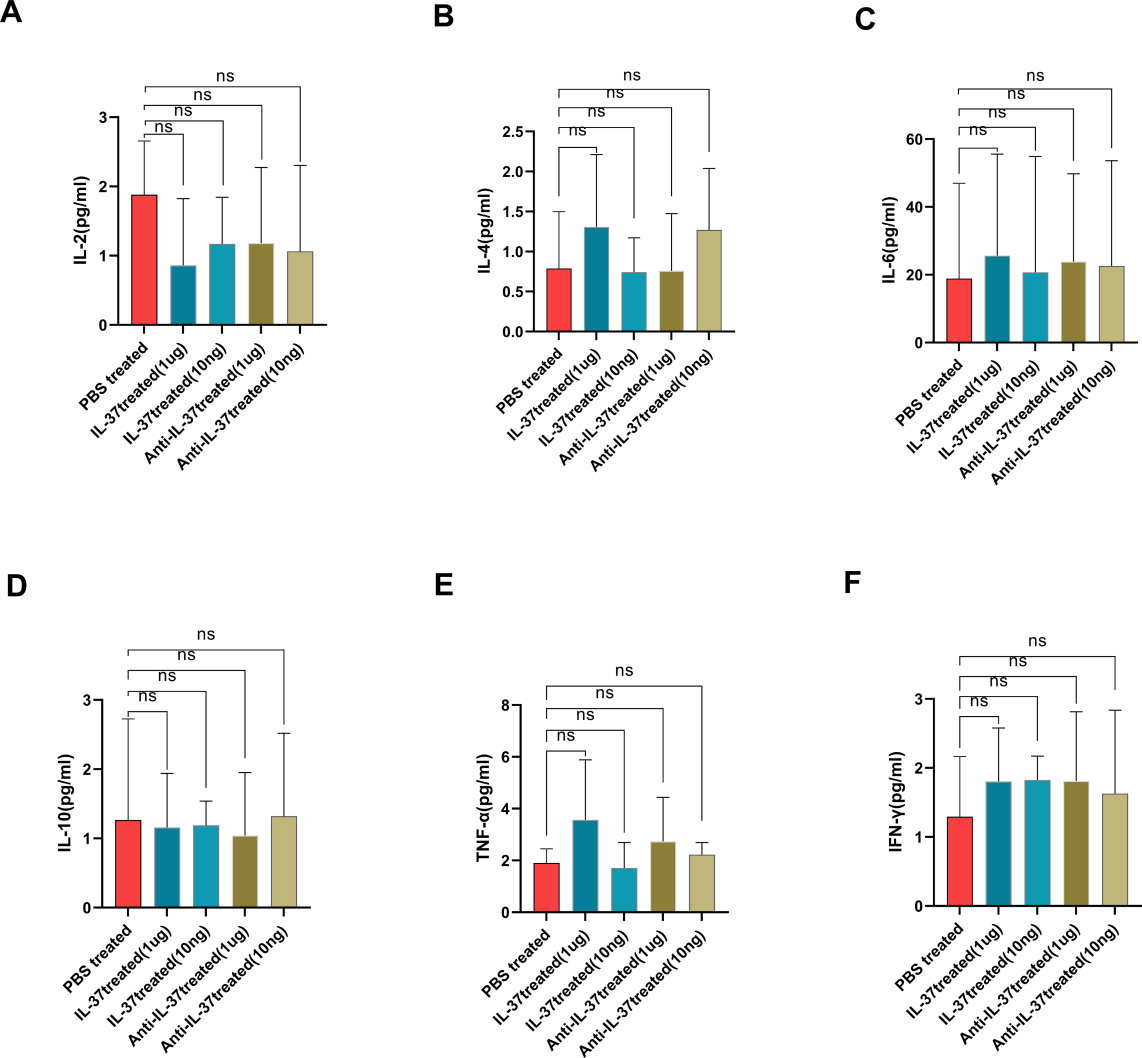
The concentrations of inflammatory cytokines IL-2, IL-4, IL-6, IL-10, TNF-α, and IFN-γ in the culture supernatant following treatment with IL-37 or PBS for 24 hours on PBMCs derived from pediatric patients with sepsis (n=4). The cytokines were quantified using the flow cytometry CBA method. **a–f** display the concentrations of IL-2, IL-4, IL-6, IL-10, TNF-α, and IFN-γ, respectively, in the culture supernatant after treatment.

**Figure 7:**
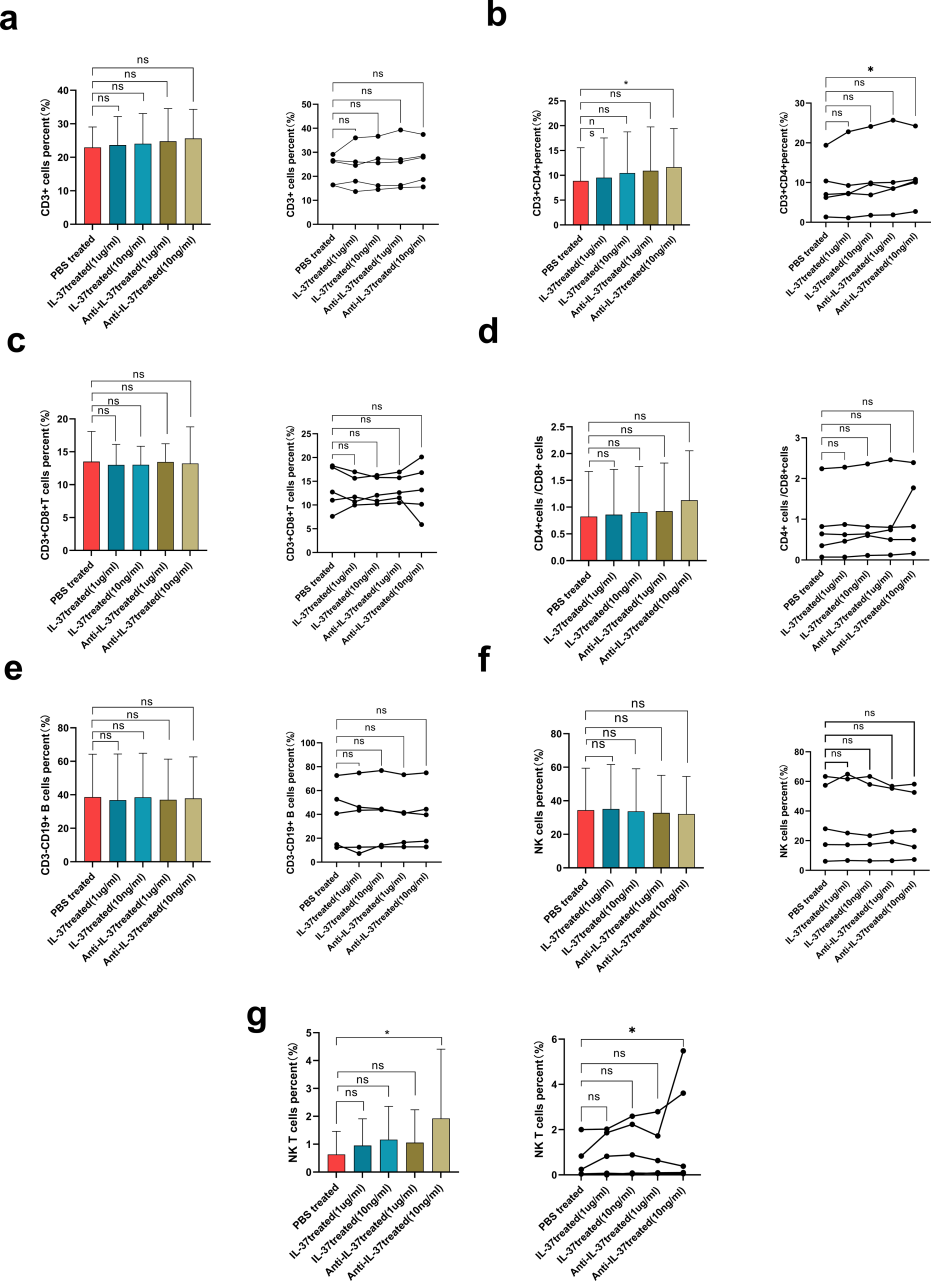
The role of IL-37 in the immune regulation of sepsis *in vitro*. It presents the changes on lymphocyte subsets in pediatric sepsis patients following the administration with IL-37 in PBMCs. The parameters measured included: **(a)** the percentage of CD3+ T cells, **(b)** the percentage of CD4+ T cells, **(c)** the percentage of CD8+ T cells, **(d)** the CD4+/CD8+ ratio, **(e)** the percentage of CD3-CD19+ B cells, **(f)** the percentage of NK cells, and **(g)** the percentage of NKT cells. A statistically significant difference was observed with *P < 0.05 when compared to pediatric septic PBMCs treated with PBS control, as determined by the Mann-Whitney U test.

PBMCs were isolated from pediatric patients with sepsis using Ficoll density gradient centrifugation. The cells were cultured for 24 hours in the presence of either recombinant human IL-37 protein (1 µg/ml, 10 ng/ml) or an anti-IL-37 neutralizing antibody (1 µg/ml, 10 ng/ml). Analysis of culture supernatants revealed no significant differences in the concentrations of inflammatory cytokines (IL-2, IL-4, IL-6, IL-10, TNF-α, and IFN-γ) between the IL-37-treated and PBS-treated groups (**Figure 6**). However, flow cytometric analysis of lymphocyte subpopulations demonstrated that neutralization of IL-37 with the anti-IL-37 antibody (at 10 ng/ml) resulted in a significant increase in the percentages of both CD4^+^ T cells and NKT cells (**Figure 7**).

## 4 Discussion

Sepsis is linked to elevated morbidity and mortality rates, particularly among pediatric patients. The pathophysiological mechanisms of sepsis are complex and involve multiple factors; however, immunosuppression has emerged as a significant contributor to mortality in this condition (30). IL-37 acts as an anti-inflammatory cytokine (31, 32), yet its clinical relevance in pediatric sepsis remains to be fully elucidated. Our current investigation suggests that IL-37 may serve as a promising biomarker and potential immunomodulatory agent for pediatric sepsis, as evidenced by three significant findings: 1) serum IL-37 levels were significantly elevated upon admission in pediatric sepsis patients, with hierarchical increases observed in cases of septic shock across two distinct cohorts; 2) IL-37 exhibited valuable adjunctive diagnostic performance, particularly when utilized alongside established biomarkers; and 3) both *in vivo* and *in vitro* experiments demonstrated that IL-37 influences inflammatory responses and immune cell activity, underscoring its potential therapeutic role in pediatric sepsis.

IL-37 levels are elevated in sepsis, resulting from the interaction of two mechanisms: resistance and defense against pathogens within the host. Bacteria are among the most common pathogens causing sepsis, with significant differences in pathogenic mechanisms between Gram-negative and Gram-positive bacteria (33). Notably, no significant differences were observed between bacterial and non-bacterial sepsis, or between Gram-positive and Gram-negative bacterial infections. This lack of variation suggests that the increase in IL-37 may be more closely associated with the common final pathway of systemic hyperinflammation and immune dysregulation in sepsis, rather than pathogen-specific components. As a broad-spectrum immunosuppressive cytokine, IL-37 functions as a universal negative regulator of immune activation, with its release correlating with the severity of the inflammatory response, regardless of the initiating pathogen type.

Accurately diagnosing sepsis early, along with effective risk stratification, remains a significant challenge due to the complex and heterogeneous nature of the condition (34). To improve patient outcomes in sepsis, it is crucial to establish reliable and timely diagnoses, manage inflammation effectively, and evaluate the immune status of patients to facilitate targeted therapies (35, 36). Existing biomarkers, such as CRP and PCT, demonstrate inadequate specificity, which limits their ability to differentiate sepsis from other inflammatory disorders (12). In this study, IL-37 exhibited promising diagnostic performance, achieving AUC values of 0.76 and 0.77 in two independent cohorts. Its independent discriminative ability is considered moderate, with performance comparable to that of C-reactive protein and interleukin-6. However, the specificity of IL-37 was found to be superior to that of both C-reactive protein and interleukin-6. Further investigations indicated that the combination of IL-37 with PCT, CRP, and IL-6 significantly enhanced diagnostic accuracy (AUC: 0.87–0.89). These findings suggest that IL-37 may serve as a valuable adjunctive biomarker.

Inflammation serves as a central element in the pathophysiology of sepsis (1, 37). The treatment of sepsis primarily focuses on infection management; however, there remains an ongoing need for innovative targeted therapeutic approaches (38). Beyond its diagnostic role, our findings suggest that IL-37 may have an immunomodulatory function in sepsis. In a murine sepsis model, the administration of recombinant IL-37 resulted in a significant reduction in systemic inflammation, decreased levels of M1 macrophages, and improved survival rates, highlighting its potential as a therapeutic agent. The protective effects of IL-37 have been previously demonstrated in models of gouty arthritis and chronic colitis (39, 40). Consistent with these findings, we observed a significant decrease in M1 macrophages in mice treated with IL-37, with no substantial changes in M2 macrophage populations or overall macrophage counts. This shift towards a less inflammatory profile is likely a key factor contributing to the improved outcomes documented in our study. An imbalance in the polarization of M1 and M2 macrophages is known to influence the onset and progression of sepsis (41). IL-37 mainly inhibits the production of pro-inflammatory cytokines by influencing macrophage polarization (42, 43), lipid metabolism, inflammasome function, TSLP synthesis, and miRNA activity. For instance, Zhou et al. demonstrated that IL-37 inhibits M1 polarization by suppressing the NF-κB and Notch1 signaling pathways (44). Similarly, Yang et al. found that IL-37 alleviates periodontitis by balancing M1 and M2 macrophages and inhibiting inflammasome activation (42). During systemic infections, M1 polarization initially dominates, leading to organ damage (45); however, sustained M1 activation can exacerbate tissue injury, prompting a shift towards M2 macrophages that promote resolution via the secretion of IL-10 and transforming growth factor-β (45). Notably, while our model demonstrated no significant change in M2 macrophage levels in response to IL-37, the elevated levels of IL-10 observed in PLF may originate from alternative immune sources. This observation suggests that the immunomodulatory influence of IL-37 extends beyond macrophages, potentially implicating T cells, B cells, or other regulatory populations.

To further explore we performed ex vivo investigations utilizing PBMCs obtained from pediatric patients with sepsis. The neutralization of IL-37 resulted in heightened percentage of CD4+ T cells and natural killer T cells (NKT), highlighting the role of IL-37 in regulating adaptive immunity. However, the absence of significant changes in cytokine levels in the supernatant—despite alterations in cellular composition—suggests the presence of complex regulatory mechanisms. T cell subsets undergo dynamic changes throughout the initiation and progression of sepsis (46). CD4+ T cells are essential for both cellular and humoral immune responses following-sepsis infection (47). Following the onset of sepsis, there is a pronounced decrease in CD4+ T cell counts (48). Conversely, research by Chen’s group indicated that CD4+ T cells were unusually elevated during the early phases of sepsis, subsequently returning to a cellular condition analogous to that of the healthy cohort two weeks after the disease began (46). This prompts an inquiry into why the proportions of CD4+ T cells and NKT cells rise post-treatment with anti-IL-37, while cytokine levels remain constant. The neutralization of IL-37 led to an expansion of CD4^+^ T and NKT cells, likely due to enhanced cellular survival or retention rather than increased effector capability, which would explain the constrained cytokine secretion. Alternatively, the expanded CD4+ T cells may represent non-cytokine-secreting subsets, such as regulatory T cells, rather than Th1/Th2/Th17 populations. As IL-37 is part of an extensive immunosuppressive network, its blockade may induce a compensatory rise in other inhibitory mediators (e.g., IL-10, TGF-β, PD-1). This homeostatic recalibration could suppress cytokine output, thereby rebalancing the system and curbing disproportionate inflammation. Moreover, the septic milieu imposes distinct metabolic and signaling adaptations on immune cells. Consequently, even with numerical increases in lymphocyte subsets, their heightened activation thresholds may necessitate co-stimulatory signals not provided in ex vivo PBMC cultures, thereby limiting cytokine detection. One potential explanation could involve the IL-37 signaling complex, which includes IL-18Rα and IL-1R8, as it hinders NF-κB and MAPK pathways while promoting anti-inflammatory signals through Mer-PTEN-DOK (17). Due to the limitations of the PBMC model, the absence of these receptors *in vitro* could disrupt functional complex formation. Finally, the transient nature of cytokine release means that the sampling timepoint may have captured a plateau or refractory phase rather than the peak. Technical limitations, such as assay sensitivity or a restricted cytokine detection panel, could also contribute to the observed discordance. Collectively, the disconnect between cellular shifts and cytokine levels highlights the complex, network-based immunoregulatory role of IL-37.

Our study provides insights into IL-37 in pediatric sepsis; however, several limitations exist. Firstly, despite including two distinct cohorts, the sample size remains relatively small. There is a need for larger, multi-center international studies to confirm the diagnostic value and general applicability of IL-37 in pediatric sepsis, necessitating a standardized rapid assay. Key steps include validating its diagnostic and prognostic value in large multicenter cohorts. Secondly, longitudinal IL-37 measurements, predictive mortality analyses, and potential unmeasured confounders (e.g., comorbidities) have yet to be developed, representing significant constraints that future research should prioritize. Additionally, while our data suggest that IL-37 modulates immune cell subsets, the specific mechanisms underlying its effects on proliferation, activation, and differentiation remain undefined. Future studies utilizing fluorescent staining, cell phosphorylation, transcription factor staining, and single-cell sequencing of IL-37 modulation could further elucidate the regulatory mechanisms of cells during sepsis progression.

In conclusion, our investigation provides initial evidence that IL-37 levels are significantly elevated in pediatric patients with sepsis and may serve as a valuable diagnostic biomarker. Through a combination of clinical cohorts, murine sepsis models, and *in vitro* immune assays, we demonstrate that IL-37 suppresses pro-inflammatory cytokines and modulates macrophage *in vivo*, while its neutralization influences T cell subsets ex vivo. Collectively, these findings reveal a critical immunoregulatory role for IL-37 in pediatric sepsis and highlight its dual promise as both a diagnostic marker and a therapeutic target.

## Data Availability

The raw data supporting the conclusions of this article will be made available by the authors, without undue reservation.
